# Unapproved peptide therapies and dermatologic care: Ethical boundaries in a gray-market era

**DOI:** 10.1016/j.jdin.2026.09.006

**Published:** 2026-09-28

**Authors:** Tarek Zieneldien, Sophia Ma, Jane M. Grant-Kels

**Affiliations:** a Johns Hopkins University School of Medicine, Baltimore, Maryland; b Department of Dermatology, University of Connecticut School of Medicine, Farmington, Connecticut; c Department of Dermatology, University of Florida College of Medicine, Gainesville, Florida

**Keywords:** ethics, gray-market therapeutics, harm reduction, motivational interviewing, online health marketing, patient autonomy, patient safety, peptide therapy, self-administration, unapproved drugs

Interest in peptide therapies in dermatology has expanded, particularly in wellness, aesthetic, and performance-enhancement markets. Although many peptide drugs have gone through rigorous approval processes evaluating their efficacy and safety, a parallel gray market of unapproved compounds has emerged, operating largely outside formal oversight.^1^ It is helpful to distinguish between research-use- only, investigational/clinical-grade, and Food and Drug Administration (FDA)-approved products. Investigational/clinical-grade peptides may be manufactured for use in clinical research but have not received FDA approval for routine clinical utilization, and research-use-only products are not intended for human use. These should also be differentiated from peptides that are legally compounded, as those are not FDA-approved and therefore do not undergo the same premarket review for efficacy, safety, and manufacturing quality. Mainstream e-commerce listings may pair “research-use only” disclaimers with recovery claims, giving patients a false sense of legitimacy.^2^

The regulatory concerns surrounding gray market peptides extend beyond the United States. In the European Union, only licensed pharmacies/authorized retailers are able to sell medicines, including online, with additional safeguards mandating: (1) imported active substances meet good manufacturing practice standards equivalent to the European Union, (2) certain medications must carry authenticity verification features, and (3) penalties be imposed for selling falsified products.^3^ Singapore similarly mandates that therapeutic products undergo regulatory review before they are sold, and the Health Sciences Authority has previously removed illegal/adulterated health products sold online.^4^

Aside from regulatory considerations, respecting a patient’s autonomy requires acknowledging a competent individual’s decision to pursue unapproved therapies. Nonetheless, if you comply with your patient’s request that you administer the peptides they obtained through online vendors, you may impart medical legitimacy to a substance whose identity, purity, pharmacokinetics, and long-term adverse effect profile are unknown. Moreover, declining to administer the peptides only satisfies one part of nonmaleficence, as counseling and harm reduction should also be pursued. These strategies include identifying the peptide/product if possible, the source, frequency, and route of administration, and discouraging high-risk acts such as sharing needles, thereby avoiding the facilitation of exposure to potentially mislabeled or biologically unpredictable compounds. Impurity profiling studies have shown that falsified and counterfeit versions of synthetic peptides have variations in terms of drugs per unit and low purities (ranging from 5% to 75% for peptides that contain cysteine) and have also been found to contain toxic elemental impurities, such as lead and arsenic.^5^ Simultaneously, beneficence requires redirecting the conversation toward the patient’s underlying goal, such as alopecia treatment, antiaging, or wound healing, and offering regulated, evidence-based alternatives.

If the patient continues to use peptides, discourage ongoing use through motivational interviewing, document discussions, examine injection sites, review warning signs (such as fever, abscess, ulceration, nodules, allergic reactions, pigmentary changes, or systemic symptoms), and arrange follow-ups rather than dismissing the patient from care. For patients who are willing to stop unregulated peptides but express concern regarding issues surrounding distributive justice, including cost, insurance coverage, or access to approved therapies, discuss lower-cost alternatives or provide resources, such as GoodRx. For hair loss, this could include generic topical or oral minoxidil when appropriate or referral to free procedural clinics.

## Conflicts of interest

None disclosed.
